# Effect of Chitosan Coatings with Cinnamon Essential Oil on Postharvest Quality of Mangoes

**DOI:** 10.3390/foods10123003

**Published:** 2021-12-04

**Authors:** Kaibo Yu, Jing Xu, Lei Zhou, Liqiang Zou, Wei Liu

**Affiliations:** 1 State Key Laboratory of Food Science and Technology, Nanchang University, Nanchang 330047, China; yukaibobj@163.com (K.Y.); 18608645197@163.com (J.X.); zouliqiang2010@163.com (L.Z.); liuwei@ncu.edu.cn (W.L.); 2 National R&D Center for Freshwater Fish Processing, Jiangxi Normal University, Nanchang 330022, China

**Keywords:** emulsion, cinnamon essential oil, edible coating, mango, postharvest quality

## Abstract

Mango (*Mangifera indica* Linn.) is a famous climacteric fruit containing abundant flavor and nutrients in the tropics, but it is prone to decay without suitable postharvest preservation measures. In this study, the chitosan (CH)-cinnamon essential oil (CEO) Pickering emulsion (CH-PE) coating was prepared, with cellulose nanocrystals as the emulsifier, and applied to harvested mangoes at the green stage of maturity. It was compared with a pure CH coating and a CH-CEO emulsion (CH-E) coating, prepared with the emulsifier Tween 80. Results showed that the CH-PE coating had a lower water solubility and water vapor permeability than the other coatings, which was mainly due to electrostatic interactions, and had a better sustained-release performance for CEO than the CH-E coating. During mango storage, the CH-PE coating effectively improved the appearance of mangoes at 25 °C for 12 d by reducing yellowing and dark spots, and delayed water loss. Hardness was maintained and membrane lipid peroxidation was reduced by regulating the activities of pectin methyl esterase, polygalacturonase, and peroxidase. In addition, the nutrient quality was improved by the CH-PE coating, with higher contents of total soluble solid, titratable acid, and ascorbic acid. Therefore, the CH-PE coating is promising to comprehensively maintain the postharvest quality of mangoes, due to its enhanced physical and sustained-release properties.

## 1. Introduction

Mango (*Mangifera indica* Linn.) is a famous fruit in the tropics, containing a variety of nutrients, such as vitamin C, organic acids, minerals, and dietary fiber [1]. Moreover, it is classified as a climacteric fruit and has an array of biochemical alterations, such as oxidative processes and the degradation of cell wall components after harvest [2]. Without effective storage, mango is prone to decay, seriously affecting its edible and commercial value. Operations which are used for preserving the postharvest quality of mangoes include cold storage, modified atmospheres, radiation storage, heat treatment, chemical treatment, etc. [3]. However, some of these operations may require expensive equipment, while others may injure the quality of mangoes and affect consumer acceptance [4]. When compared with other postharvest technologies, edible coatings have many advantages. They can form a protective layer on the surface of the mangoes, which can effectively block the epidermal pores and regulate the gas microenvironment around the mangoes, thus reducing water transpiration and oxidation caused by the amount of oxygen transmission, and maintaining the quality of mangoes [5,6].

Chitosan (CH), a cationic polysaccharide obtained from the deacetylation of chitin, has been studied as a fruit-coating material, due to its non-toxicity, biodegradability, and film-forming ability [7]. Nevertheless, CH is not an ideal coating material, due to its hydrophilicity and poor water–vapor barrier properties [8]. Some studies have shown that the lipophilic properties of some components in essential oils can change the water solubility and water vapor permeability of CH coatings, which reduces respiration rate of fruits and delays fruit ripening [9,10,11]. Essential oils are aromatic substances that are extracted from different tissues of plants, such as the flowers, leaves, stems, roots, or fruits. They are widely used in the food industry due to their good antioxidant, antibacterial, and antifungal effects [12]. Cinnamon essential oil (CEO) is usually a yellow to reddish-brown clear liquid with the unique pungent aroma of Chinese cinnamon. CEO has been proven to be an excellent antioxidant and has a broad-spectrum antimicrobial effect, with its main component being cinnamaldehyde [13,14]. Unfortunately, the use of CEO has been limited, due to its instability and poor water solubility. In order to solve these problems, it is important to develop a novel CH-based active coating for preserving mangoes, by selecting appropriate encapsulation technology, which can also better exert the efficacy and mask the odors of CEO.

Pickering emulsion is a type of emulsion that is stabilized by solid particles instead of surfactants as emulsifiers, which can effectively encapsulate hydrophobic components [15]. When compared with traditional emulsions, it has the advantages of having less emulsifier consumption, a low cost, good stability, and environmental friendliness. Cellulose nanocrystals (CNC), a sustainable nanomaterial, are the crystalline portion of cellulose microfibrils and are considered as ideal biological materials due to their excellent properties (such as a high aspect ratio and exceptional mechanical properties) [16]. CNC can be used as a filler to reinforce other polymer matrices, as well as a Pickering agent to form mechanical (steric) barriers in oil/water interfaces, to protect emulsion droplets against coalescence [17]. Deng et al. [18] successfully prepared a Pickering emulsion using oleic acid as the oil phase and CNC as the stabilizer, which effectively delayed the ripening of Bartlett pears.

At present, there are few reports about the use of CH-based Pickering emulsion coatings to maintain the postharvest quality of mangoes. In this study, the CH-CEO Pickering emulsion (CH-PE) coating, prepared with CNC, was characterized and compared with pure CH coating and CH-CEO emulsion (CH-E) coating, prepared with Tween 80. Moreover, the coatings were applied to fresh mangoes, and the quality and physiological indices of mangoes were measured to compare their fresh-keeping effects on mangoes.

## 2. Materials and Methods

### 2.1. Materials

Mangoes (*Mangifera indica* Linn. *Tainung*) were purchased from a commercial mango base (Hainan, China). They were selected according to their uniformity in size (approximately 80 g for each mango), color (green), and ripeness (at 75–80%, commercial maturity stage), and without mechanical damage or any visible defects. CEO was purchased from Yueyu Essential Oil Mall (Guangdong, China). CH, guaiacol, 3,5-dinitrosalicylic acid and pectin were purchased from Aladdin Chemicals Co. (Shanghai, China). D-(+)-galacturonic acid, bromothymol blue, and thiobarbituric acid were obtained from Beijing Solarbio Science and Technology Co., Ltd. (Beijing, China). 2,6-dichlorophenolindophenol and catechol were purchased from Macklin Chemicals Co. (Shanghai, China). The other chemicals used in this study were analytical grade and were purchased from domestic reagent companies.

### 2.2. Preparation of Coating Solutions

An amount of 10 g of CH powder was dissolved in 500 mL of 1% aqueous acetic acid solution (*v*/*v*). For preparing the CNC Pickering emulsion, 0.4% CEO (*v*/*v*) was added into 0.2% CNC aqueous suspensions (*w*/*v*) and then sheared at 12,000 rpm for 3 min using a high-shear disperser (ULTRA TURRAX^®^ T18, IKA, Staufen, Germany). The CNC Pickering emulsion was mixed with an equal amount of 2% CH solution (*w*/*v*) and the mixture was sheared at 12,000 rpm for 1 min. The mixed solution was represented by CH-PE. Meanwhile, the emulsion prepared by the commonly used emulsifier Tween 80 was selected for comparison. A total of 0.4% CEO (*v*/*v*) was added into the aqueous solution containing 0.2% Tween 80 (*w*/*v*) and then sheared at 12,000 rpm for 3 min. The emulsion was mixed with an equal amount of 2% CH (*w*/*v*) and the mixture was sheared at 12,000 rpm for 1 min. The mixed solution was represented by CH-E. Below are the information and the labels of the samples in each group.

Control: distilled water;
E: 0.1% Tween 80 (*w*/*v*) + 0.2% CEO (*v*/*v*);
PE: 0.1% CNC (*w*/*v*) + 0.2% CEO (*v*/*v*);
CH: 1% CH (*w*/*v*);
CH-E: 1% CH (*w*/*v*) + 0.1% Tween 80 (*w*/*v*) + 0.2% CEO (*v*/*v*);
CH-PE: 1% CH (*w*/*v*) + 0.1% CNC (*w*/*v*) + 0.2% CEO (*v*/*v*).

### 2.3. Electrical Charges of the Emulsions

The electrical charges of the emulsions and CH solution before and after mixing were measured using a particle electrophoresis instrument (Zetasizer Nano ZSP, Malvern Instruments, Worcestershire, UK).

### 2.4. Characterization of Edible Coatings

The coating solutions were cast on the glass plates and dried completely at room temperature. The dried coatings were carefully peeled and stored in a 50% relative humidity (RH) dryer at 25 °C for 48 h before measuring.

#### 2.4.1. Water Solubility (WS)

The coating sample (20 mm × 20 mm) was dried in an oven at 105 °C for 2 h to obtain the dry coating weight (*m*_0_). The dried coating was placed in small glass containers containing 30 mL distilled water at room temperature for 24 h. After filtering with filter paper, the undissolved coating was obtained. The filtered coating was dried in an oven at 105 °C for 2 h and weighed (*m*_1_). The WS of the coating was calculated by the following formula:

(1) $$\mathrm{WS}\;\left(\%\right)=\frac{m_{0}-m_{1}}{m_{0}}\times 100\%$$

#### 2.4.2. Water Vapor Permeability (WVP)

The water vapor transmission rates (WVTR) of the samples were measured using the PERMATRAN-W^®^ Model 150 (Mocon, Minneapolis, MN, USA), which was according to ASTM E398 via dynamic RH measurement. The instrument was calibrated at 37.8 °C with a NIST-certified Mylar film that had known water vapor transport characteristics. The measurements were carried out at 37.8 °C with a 30% RH gradient for all samples. The WVP was calculated by using the following formula:

(2) $$\mathrm{WVP}=\frac{\mathrm{WVTR}\times L}{P^{sat}\times \Delta RH}$$

where *L*, *P^sat^*, Δ*RH* (%) are coating thickness, water vapor saturation pressure at 37.8 °C, and percentage of the RH gradient, respectively.

#### 2.4.3. Migration of CEO in Different Food Simulants

According to the method described by Lian et al. [19], with slight modifications, distilled water, 50% ethanol, and 95% ethanol were selected as standard food simulants. At room temperature, the coating sample (20 mm × 20 mm) was immersed in a centrifuge tube containing 50 mL of simulated food solution. Samples were taken at regular intervals for measurement. After each measurement, the simulated solution was added to the immersion solution again to the specified scale. The final solution was measured by an ultraviolet-visible spectrophotometer (UV-1600PC, Mapada, Shanghai, China), with the absorbance at 286 nm. Before testing, full-band scanning was used to determine the maximum absorption peak of CEO, and a standard curve was produced.

### 2.5. Application of Coating in Mango Fresh Storage

The harvested mangoes, with the same maturity and size, were transported to the laboratory. The prepared mangoes were randomly divided into four groups of 36 mangoes each and 9 mangoes were prepared for the analysis of the original mangoes. Mangoes were immersed in 0.3% sodium hypochlorite solution (*v*/*v*) for 5 min, rinsed, dried, and then were separately subjected into distilled water (control group) and three coating solutions (CH, CH-E and CH-PE) for 3 min. Subsequently, the mango samples were ventilation dried at room temperature for 2 h. Samples were then stored at 25 ± 1 °C for 12 d. Three mangoes were randomly taken from each group every 3 d, and combined or mixed by a small juicer to assess the quality attributes of the mangoes with different treatments, which was carried out three times for replication.

### 2.6. Weight Loss

The weight loss was determined by weighing the samples before and after the storage period, which were directly exposed to 25 ± 1 °C and 60% ± 3% RH (ambient conditions). It was expressed as the percentage of weight loss compared to the initial weight.

### 2.7. Hardness

The hardness of the mangoes was determined based on the method of a previous study, with some modifications [20]. The mangoes were cut into 2 cm × 2 cm × 1.2 cm cubes. The hardness of the mangoes was determined by using a TA-XT plus texture analyzer (Stable Micro Systems, Godalming, UK) and a P5 probe (pre-measurement speed = 1.00 mm/s, measurement speed = 0.5 mm/s, post-measurement speed = 1.00 mm/s and compression ratio = 35%).

### 2.8. Total Soluble Solid (TSS)

The TSS content was measured according to the method of Yin et al. [21]. A 25 g mass of peeled mango pulp was mashed in a juicer and filtered through gauze. The TSS content of the juice was measured using a digital Abbe refractometer.

### 2.9. Titratable Acidity (TA)

TA was measured according to the method of Zhang et al. [22], with some modifications. An amount of 10 g of the well-mixed mango sample was taken and transferred to a 100 mL volumetric flask, allowed to stand for 30 min, and then filtered. An amount of 20 mL of the filtrate was treated by adding two drops of 1% phenolphthalein indicator and titrated with 0.1 mol/L sodium hydroxide standard solution. TA was calculated according to the following formula:

(3) $$\mathrm{TA}\;\left(\%\right)=\frac{V_{0}\times N\times 0.064\times V_{1}}{m\times V_{2}}\times 100\%$$

where *V*_0_ is sample volume, *N* is sodium hydroxide solution concentration, 0.064 is the conversion factor calculated using citric acid, *V*_1_ is the volume of sodium hydroxide solution that was consumed by the titration of the filtrate, *m* is the sample weight, and *V*_2_ is the filtrate volume that was used for titration.

### 2.10. Ascorbic Acid Content

The ascorbic acid content was determined according to the method of 2,6-dichlorophenolindophenol titration. Thirty grams of mango pulp and 30 mL of 2% metaphosphoric acid solution were mixed with a juicer. Then 30 g of the homogenized sample was transferred to a 100 mL volumetric flask with 2% metaphosphoric acid solution. The filtrate plus the kaolin decolorizer were mixed and filtered. Next, 10 mL of the filtrate was titrated with the calibrated 2,6-dichlorophenolindophenol solution. The ascorbic acid content was calculated using the following formula:

(4) $$\mathrm{Ascorbic}\;\mathrm{acid}\;\mathrm{content}\;\left(\mathrm{mg}/100\;g\right)=\frac{\left(V-V_{0}\right)\times T\times A}{W}\times 100$$

where *V* is the volume of the 2,6-dichlorophenolindophenol solution consumed by the titration sample, *V*_0_ is the volume of the 2,6-dichlorophenolindophenol solution consumed by the titration blank, *T* is the 2,6-dichlorophenolindophenol solution titer, *A* is the dilution factor, and *W* is sample weight.

### 2.11. Malondialdehyde (MDA) Content

The MDA content was measured as described by Yang et al. [23]. One gram of mango tissue was homogenized with 5 mL of 10% trichloroacetic acid and then centrifuged for 20 min at 10,000× *g*. Furthermore, 1.5 mL of the supernatant was mixed with 2.5 mL of 0.67% thiobarbituric acid, which was previously dissolved in 0.05 mol/L sodium hydroxide solution. The reaction solution was heat-treated for 20 min at 95 °C, cooled rapidly, and then centrifuged for 10 min at 10,000× *g*. The absorbance of the supernatant was measured at 600, 532, and 450 nm, respectively. The MDA content in the mixture was calculated according to the following formula, which can be converted to the MDA content in each gram of mango pulp.

(5) $$\mathrm{MDA}\;\mathrm{content}\;\left(\mu \mathrm{mol}⁄L\right)=6.45\times \left(OD_{532}-OD_{600}\right)-0.556\times OD_{450}$$

### 2.12. Polyphenol Oxidase (PPO) and Peroxidase (POD) Activity Assay

The extraction and activity assays of the mango PPO and POD were conducted according to Zhou et al. [24], with some modifications. The enzyme extraction solution was 0.1 mol/L phosphate buffer (pH 5.5) containing 1 mol/L sodium chloride, 2% polyvinylpolypyrrolidone (*w*/*v*), and 1% Triton X-100 (*v*/*v*). An amount of 30 g of mango sample was mixed with 30 mL enzyme extraction solution. After homogenizing for 3 min, the mixture was centrifuged at 11,000× *g* for 20 min at 4 °C. The supernatant was used as a crude enzyme extract for the PPO and POD activity assay.

For the PPO activity assay, the reaction mixture consisted of 0.2 mL of enzyme extract and 3.2 mL of phosphate buffer (0.1 mol/L, pH 4.5) containing 0.06 mol/L catechol. The absorbance was monitored at 420 nm for 1 min, and one unit of PPO activity was defined as the amount of enzyme that caused an increase of 0.01 in the absorbance per minute.

For the POD assay, the reaction mixture contained 0.1 mL of enzyme extract, 0.5 mL of 0.025 mol/L guaiacol, 0.2 mL of 0.5 mol/L hydrogen peroxide solution, and 2.7 mL phosphate buffer (0.1 mol/L, pH 4.5). The absorbance was monitored at 485 nm for 5 min, and one unit of POD activity was defined as the amount of enzyme that caused an increase of 0.01 in the absorbance per minute.

### 2.13. Phenylalanine Ammonialyase (PAL) Activity Assay

The PAL activity was measured using the PAL kit, produced by Nanjing Jiancheng Bioengineering Institute (Jiangsu, China). An amount of 1 g of pulp tissue and 9 mL of extraction buffer were mixed and ground under an ice bath. Then the homogenate was centrifuged at 10,000× *g* for 20 min at 4 °C and the supernatant was prepared for the enzyme activity determination. The measurement procedure referred to the product specification, and the absorbance of the reaction solution was measured at a wavelength of 290 nm. One unit of PAL activity was defined as the amount of enzyme that caused an increase of 0.01 in the absorbance per minute.

### 2.14. Pectin methyl Esterase (PME) Activity Assay

The activity of PME was determined according to the method of Fan et al. [25], with slight modifications. Four grams of mango was ground with 8.8% sodium chloride solution (*w*/*v*), followed by shaking for 4 h and centrifuging for 20 min. The supernatant was collected as PME extract. An amount of 0.4 mL of the extract was mixed with 0.7 mL of distilled water, 2.0 mL of pectin solution, and 0.3 mL of bromothymol blue. The absorbance of the reaction solution was determined at 620 nm. One unit of PME activity was defined as the amount of enzyme that caused a change of 0.01 in absorbance at 620 nm per minute.

### 2.15. Polygalacturonase (PG) Activity Assay

Ten grams of mango was homogenized in a pre-cooled mortar with 20 mL of pre-cooled 95% ethanol, then was left at low temperature for 10 min, and then centrifuged at 12,000× *g* for 20 min at 4 °C. The supernatant was poured off. An amount of 10 mL of pre-cooled 80% ethanol was added to the precipitate, which was left at a low temperature for 10 min and centrifuged at 12,000× *g* for 20 min at 4 °C. Then, the supernatant was decanted, and 5 mL of pre-cooled sodium acetate buffer, containing 10% sodium chloride, was added to the precipitate, which was left at 4 °C for 20 min to extract the enzyme. After centrifugation, the supernatant was collected as the enzyme extract. The PG activity was determined by the 3,5-dinitrosalicylic acid colorimetric method. One unit of PG activity was expressed as the amount of enzyme that produced 1 μg free galacturonic acid per hour.

### 2.16. Data Analysis

All experiments were carried out three times and the results were expressed as the mean ± standard deviation (SD). The data were analyzed by a one-way analysis of variance (ANOVA), using SPSS Statistics 25.0 (IBM, Chicago, IL, USA). Differences between means were compared using the Student’s *t*-test or the Tukey’s Honestly Significant Difference (HSD) multiple range test, and the results were regarded as significantly different at *p* < 0.05.

## 3. Results and Discussion

### 3.1. Zeta-Potential of Emulsions and Physical Properties of Coatings

It can be seen from Table 1 that the zeta-potential of the cinnamon essential oil (CEO) Pickering emulsion (PE), prepared with cellulose nanocrystals (CNC) as the stabilizer was −55.133 mV, and the zeta-potential of the chitosan (CH) solution was +34.033 mV. The zeta-potential of the CH-CEO Pickering emulsion (CH-PE), obtained by mixing Pickering emulsion and CH solution, was +56.000 mV, which may be due to the introduction of -NH^3+^ groups on CH, neutralized the SO^3−^ group on the CNC surface [18]. The water solubility (WS) of coatings is an important factor for maintaining the freshness of fruits and vegetables. It was implied from Table 1 that the WS of coatings significantly decreased from 31.829 to 17.463 and 13.911%, due to the addition of different emulsions. It shows that the addition of hydrophobic CEO could reduce the WS of CH coating [26]. However, the emulsion stabilized by CNC could reduce WS of the coating more strongly than the emulsion stabilized with surfactant Tween 80. Water vapor is an important environmental factor affecting the quality of fruits and vegetables. The coatings that are formed on the surface of fruits and vegetables are to reduce the water transfer between fresh products and surrounding medium. Therefore, attention should be paid to the water vapor barrier properties of edible coatings [27]. As shown in Table 1, the water vapor permeability (WVP) of CH coating was 1.1869 × 10^−10^ g/m·s·Pa. The addition of CEO significantly reduced the WVP of CH coating. Comparing the CH-CEO emulsion (CH-E) coating with the CH-PE coating, it can be found that the CH-PE coating has a lower WVP (5.222 × 10^−11^ g/m·s·Pa), which may be due to the good biocompatibility between PE and the CH matrix. In conclusion, the CNC Pickering emulsion was incorporated into the CH solution to form a stable coating with good properties.

### 3.2. Migration of CEO in Different Food Simulants

**Figure 1:**
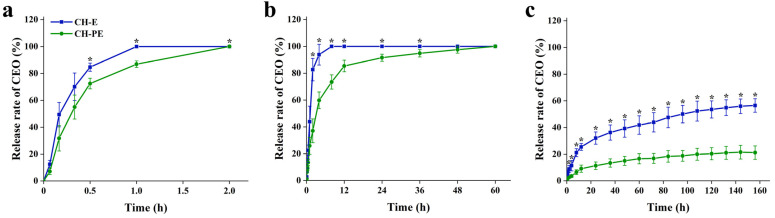
Release rates of cinnamon essential oil (CEO) from CH-E and CH-PE coatings in distilled water (**a**), 50% ethanol (**b**) and 95% ethanol (**c**). Error bars indicate the standard deviation (SD) of three replicates. Asterisks indicate statistical significance (*p* < 0.05) between CH-E and CH-PE coatings at same time using the Student’s *T*-test.

Food properties have a certain influence on the release of essential oils. The degree of release of CEO in the three different food simulants (distilled water, 50% ethanol, and 95% ethanol) are clearly reflected in Figure 1. CEOs in the CH-E and CH-PE coatings released oil the fastest in distilled water, which were completely released after 1 h and 2 h, respectively. The CEO in the CH-E coating, in 50% ethanol, reached equilibrium after 8 h, while in the CH-PE coating CEO was released completely after 60 h. When the food simulant was 95% ethanol, the release of CEO was the slowest, and the release of CEO in the CH-PE coating was still lower than that in the CH-E coating. During the release of CEO, water molecules in the simulated liquid gradually enter the CH matrix, causing the CH coating to swell and dissolve, and CEO is gradually released from the CH coating [28]. As time goes by, the coating structure becomes looser and the main force between the CH matrix is destroyed, resulting in a sharp increase in the release of CEO [19]. The fastest release of CEO in pure water might be due to the rapid hydration of the coatings in pure water. As the concentration of ethanol increased, the polarity decreased, and the release of CEO in the coatings slowed down. The slower the release rate of CEO from the polymer was, the better the stability of the composite coating was. The results showed that the CH-PE coating had a better sustained-release effect than that of the CH-E coating in different simulation fluids, indicating that CNC as an emulsifier was better than the surfactant Tween 80 in improving the performance of CH coating.

### 3.3. Effects of The Coatings on Mango Sensory and Nutrient Qualities

#### 3.3.1. Visual Appearance

**Figure 2:**
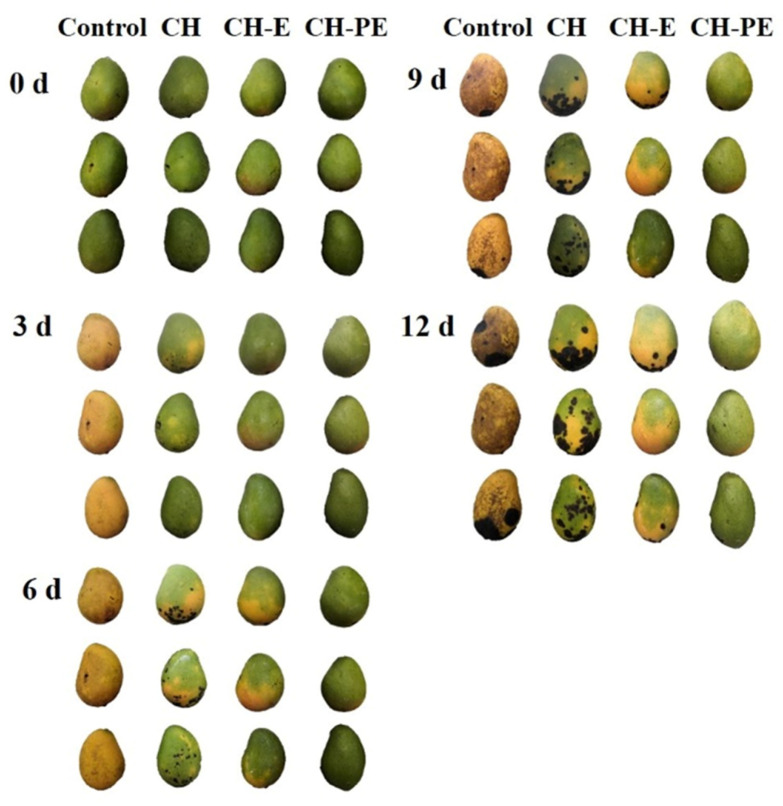
Changes in visual appearance during mango storage.

The obvious changes of the mangoes during storage were that peels turned yellow and dark spots appeared on the surface of the peels. Figure 2 shows the appearance changes of the mangoes stored at 25 °C for 12 d after different treatments. On day 3, the peels of the control group turned yellow and dark spots began to appear. The treatment groups could significantly delay the yellowing of mango peels. Mangoes which were treated with the CH coating alone turned yellow slightly on day 3, and dark spots appeared on day 6, which increased with the extension of the storage time. The mangoes which were treated with the CH-E coating turned yellow slightly on day 6, and small dark spots appeared on day 9. However, the mangoes coated with the CH-PE coating had slight yellowing and few visible dark spots on day 12. These results indicated that the CH-PE coating can not only delay the yellowing of mangoes, but also reduce dark spots on the surface of mangoes. Similarly, Ma et al. [29] reported that the composite coating based on shellac and an active agent, tannic acid, effectively reduced yellowing and dark spots, which further improved the postharvest quality and consumer acceptability of mangoes.

#### 3.3.2. Weight Loss and Hardness

**Figure 3:**
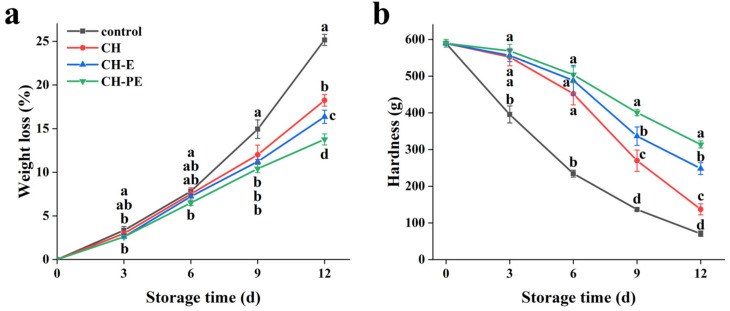
Changes in weight loss (**a**) and hardness (**b**) during mango storage. Error bars indicate the standard deviation (SD) of three replicates. Different lowercase letters indicate statistical significance (*p* < 0.05) between four groups at same time by the Tukey’s Honestly Significant Difference (HSD) test.

The transpiration of water and postharvest respiration leads to the loss of mango quality during storage [30]. The effects of the different treatments on the weight loss of mangoes are shown in Figure 3a. As the storage time increased, the weight loss of all groups increased to different degrees. From 0 d to 6 d, the weight loss of all groups were only slightly different. At this time, the weight loss of the control mangoes was 7.81%, and for the mangoes coated with the CH and CH-E coatings, the weight loss was 7.52 and 7.24%, respectively. However, the weight loss of the mangoes coated with the CH-PE coating was 6.50%. On day 12, the uncoated mangoes showed a greater weight loss of 25.16%, while the mangoes coated with the CH, CH-E, and CH-PE coatings reached 18.24, 16.36, and 13.76%, respectively. These changes indicated that the coatings could reduce the volatilization of water vapor, and thus slowed down the respiratory rate of mangoes. The weight losses for the mangoes which were coated with CH, CH-E, and CH-PE coatings were compared, and it was found that the mangoes which were treated with CH-PE coating had the lowest weight loss. This might be because the CH-PE coating has the lowest WVP and formed a dense structure, which effectively covered the cracks and pores on the mangoes’ surface and formed a mechanical barrier to water [5]. A previous study also confirmed that the polysaccharide-based coating which were incorporated with galangal essential oil had better effects on resisting water loss and maintaining the quality of mangoes than single polysaccharide coating [31].

The effects of the edible coatings on the hardness of mangoes are depicted in Figure 3b. During the 12 d of storage, the hardness of the control mangoes decreased sharply from 589.379 g to 70.792 g, indicating that the mangoes had lost their edible value, and would not be accepted by consumers. From 0 d to 6 d, the hardness of the mangoes coated with the CH, CH-E, and CH-PE coatings decreased slightly, without being significantly different. On day 12, the hardness of mangoes coated with the CH, CH-E, and CH-PE coatings were 137.150, 248.143, and 313.83 g, respectively. Due to the poor coating performance of CH alone, the hardness of the mangoes decreased rapidly in the later stage. The mangoes treated with the CH-PE coating maintained the highest hardness value during the later stage of storage, which may be due to the CH-PE coating having a low WVP and WS, which reduced water loss and the physiological activity of the mangoes [31,32]. Vázquez-Celestino et al. [20] indicated that the use of the water vapor barriers (waxing) reduced hardness loss of mangoes more effectively than blockers of ethylene action (1-MCP) or inhibitors of ethylene biosynthesis (NO).

#### 3.3.3. TSS, TA, Ascorbic Acid and MDA

**Figure 4:**
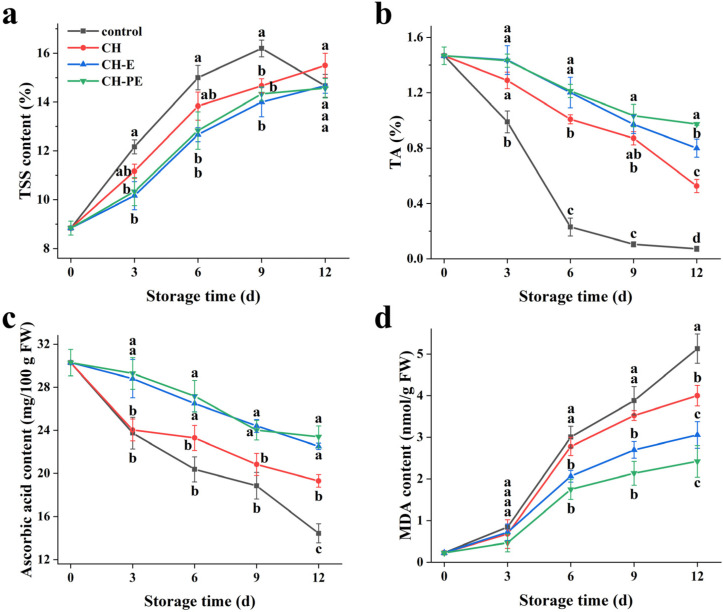
Changes in total soluble solid (TSS) content (**a**), titratable acidity (TA) (**b**), ascorbic acid content (**c**) and malondialdehyde (MDA) content (**d**) during mango storage. Error bars indicate the standard deviation (SD) of three replicates. Different lowercase letters indicate statistical significance (*p* < 0.05) between four groups at same time by the Tukey’s HSD test.

The total soluble solid (TSS) content, expressed as a percentage, is an important indicator that reflects the nutritional value and storage ability of fruits. As shown in Figure 4a, in the first 9 d of storage, the TSS contents of each group continued to increase, which might be because the respiration of mangoes decomposed a large amount of their organic matter into sugar, acid, and minerals. Subsequently, the TSS content of the uncoated mangoes began to decline due to decay and senescence of the mangoes, while the TSS contents of the coating treatment groups continued to increase. In addition, the TSS contents of the treatment groups was always lower than the highest value (16.20%) of the control group, and the TSS content of mangoes that were coated with the CH-PE coating was the lowest. The CH-PE coating on the surface of mangoes delayed the increases in TSS content and maintained the quality of mangoes.

Titratable acidity (TA) is composed of a variety of organic acids, which has an important influence on fruit flavor and storage quality. The TA of all samples showed a downward trend during storage (Figure 4b). This may be because the organic acids in mangoes were consumed by physiological effects, such as respiration and metabolism. The TA of the control group decreased rapidly from 0 d (1.47%) to 6 d (0.23%), and the TA was only 0.074% on day 12. When compared with the control group, the coating groups significantly slowed the decreases in theTA value. On day 12, the TA of the CH, CH-E, and CH-PE coating treatment groups were 0.53, 0.80, and 0.97%, respectively. Further comparison of the treatment groups found that the CH-PE coating could best maintain the TA of mangoes. The results showed that the CH-PE coating had a good inhibitory effect on mango metabolism, which inhibited the degradation of organic acids and slowed down their rate of decline.

Ascorbic acid is an important indicator for judging the nutritional value of fruits. However, ascorbic acid is easily oxidized and decomposed during storage, and its content within the mangoes significantly reduced in this study (Figure 4c). The ascorbic acid content of the control group decreased the fastest, from 30.28 to 14.42 mg/100 g during storage. However, the ascorbic acid contents of the coating treatment groups were significantly higher than that of the control group. The micro-atmospheric environment which formed on the surface of the coatings reduced the permeability of oxygen, thereby reducing the activity of oxidases inside the fruits, and delaying the oxidation of ascorbic acid [8]. In addition, the contents of ascorbic acid in the CH-E and CH-PE coating groups were the highest than the other groups after 12 d of storage. The reason might be that the release of CEO in the coating further inhibited the oxidative decomposition of ascorbic acid [21]. Thereby, the coating treatments can effectively maintain ascorbic acid content in mangoes and reduce the loss of mango nutrient quality.

Malondialdehyde (MDA) is one of the most important products of membrane lipid peroxidation during plant senescence, and is often used as an indicator for senescence evaluation [33]. A continuous increase was observed in the MDA content of the control group and the coating-treated groups during storage (Figure 4d). On day 12, the MDA content of the control group was 5.13 nmol/g, and the MDA content in the CH-PE coating-treated mangoes was only 47.24% of the control. This may be related to the coating potentially inducing the oxidation defense system, and reducing the degree of membrane lipid peroxidation, which was supported by Onik et al. [33] and Xing et al. [34].

Various studies have reported the effects of chitosan-based coatings on maintaining nutrient quality and delaying the senescence of mangoes [3,5]. The application of polysaccharide-based coatings in combination with essential oils was an innovative strategy to maintain the postharvest quality of fruits [9]. In this study, the CH-PE coating has the greatest effect when compared to the CH and CH-E coatings on maintaining mango quality. Jung et al. [35] also proposed that Pickering emulsion coating, composed of oleic acid, CNC and CH, significantly reduced ethylene production and delayed the ripening of ‘Bartlett’ pears during their long-term cold storage. It was reported that the chitosan/titanium dioxide nanocomposite coating reduced the TSS and MDA content of mangoes, which played a significant role in preserving their nutrients and quality [34]. Zhou et al. [31] indicated that a polysaccharide-based coating, incorporated with galangal essential oil, effectively increased TA and reduced the TSS content.

### 3.4. Effects of the Coatings on Activities of Mango PPO, POD and PAL

**Figure 5:**
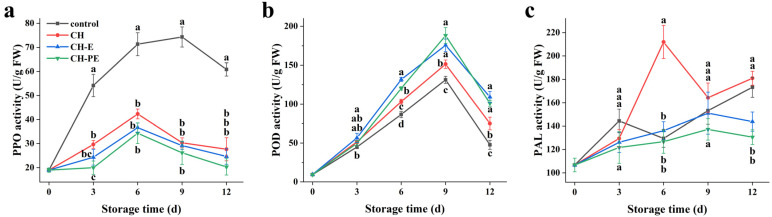
Changes in activities of polyphenol oxidase (PPO) (**a**), peroxidase (POD) (**b**) and phenylalanine ammonialyase (PAL) (**c**) during mango storage. Error bars indicate the standard deviation (SD) of three replicates. Different lowercase letters indicate statistical significance (*p* < 0.05) between four groups at same time by the Tukey’s HSD test.

Polyphenol oxidase (PPO) can catalyze the oxidation of endogenous polyphenols into quinones, and then polymerize them into melanin, which will deteriorate the color and quality of fruits and vegetables [36]. The PPO activities of all samples first increased and then decreased, but that of the coating treatment groups consistently maintained PPO activity at a relatively low level (Figure 5a). This showed that the coatings could inhibit the activity of PPO in mangoes, and the CH-PE coating had the best effect. This may be because the CEO in the CH-PE coating affected the PPO activity in mangoes. Our previous study found that the cinnamaldehyde in CEO can effectively inhibit the PPO activity in apple juice [37]. In addition, high concentrations of organic acids might decrease the activity of PPO in this substrate [2]. As can be seen from Figure 4b, the mangoes treated with the CH-PE coating had the highest TA value. Therefore, proper coating treatment can not only effectively inhibit the increase of PPO activity, and reduce the probability of fruit enzymatic browning, but also improve the storage quality of the fruit.

Peroxidase (POD), a complex enzyme in fruits, could help to reduce the oxidative damage in the regeneration of ascorbate and glutathione metabolites [11]. The POD activities of the untreated and coating-treated mangoes first increased and then decreased during storage (Figure 5b). On the ninth day of storage, the POD activities of each group reached their peak values, but the POD activities of the coating-treated mangoes were higher than that of the untreated mangoes. Among them, the CH-E and CH-PE coating-treated mangoes had the highest POD activity, indicating that the addition of CEO in the coating could better improve POD activity in mangoes, and reduce damage by reactive oxygen species to cells. These findings corresponded with the results reported by Xing et al. [34], which indicated that CH/titanium dioxide nanocomposite coating treatment enhanced the POD activity of mangoes.

Phenylalanine ammonialyase (PAL) is the first rate-limiting enzyme in the phenylpropanoid pathway, and is involved in the biosynthesis of phenolics, phytoalexins, and lignins [38]. As can be seen from Figure 5c, the PAL activities of the untreated and CH coating-treated mangoes first increased, decreased, and then increased. Moreover, the PAL activity of the CH coating-treated mangoes reached its maximum value on day six. The PAL activities in the CH-E and CH-PE coating-treated samples increased first, and then decreased, reaching their maximum values on day nine. The PAL activity in the CH-PE coating-treated mangoes was the lowest when compared with the other groups during the whole storage period. In particular, the results showed that the addition of CEO did not increase the PAL activity of mangoes. Factors such as decay, disease, and oxidative stress in the untreated and CH coating-treated mangoes during storage initiated the increase of PAL activity [12]. However, the mangoes treated with the coatings containing CEO, especially the CH-PE coating, had a much lower disease incidence during the entire storage period than the other groups, so the main disease-resistant enzyme PAL activity was weakly stimulated and increased. Similarly, Chen et al. [39] found that *Ficus hirta* Vahl, a fruit extract-incorporated chitosan coating, increased the PAL and POD activity and decreased the PPO activity, to reduce postharvest loss and enhance the storability of Xinyu tangerines.

### 3.5. Effects of The Coatings on Activities of Mango PME and PG

**Figure 6:**
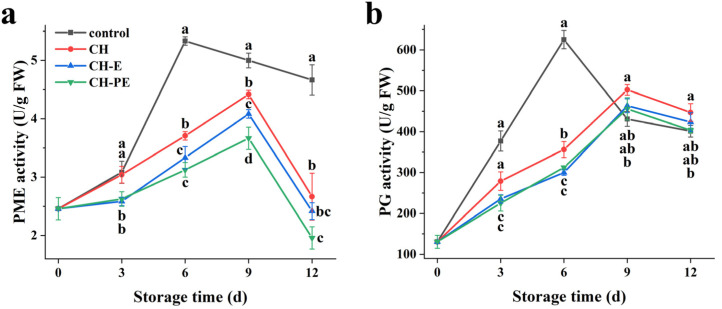
Changes in activities of pectin methyl esterase (PME) (**a**) and polygalacturonase (PG) (**b**) during mango storage. Error bars indicate the standard deviation (SD) of three replicates. Different lowercase letters indicate statistical significance (*p* < 0.05) between four groups at same time by the Tukey’s HSD test.

Softening is a complex process, which is mainly due to the changes in cell wall structure and decomposition of different cell wall components [40]. During the ripening process of the fruit, demethylation of pectin by pectin methyl esterase (PME) helps polygalacturonase (PG) to hydrolyze the α-(1-4) galacturonic acid bond of pectic acid, thus disintegrating the colloid layer in the cell wall and leading to fruit softening [41,42]. As shown in Figure 6, the PME and PG activity showed similar changes during the whole storage process, increasing first and then decreasing. The PME and PG activities of the control group reached their highest on the sixth day, and those of the coating treatment groups reached their peaks on the ninth day. At the same time, the PME and PG activity in the CH-PE coating treatment group remained the lowest when compared to the other groups, which was similar to the previous results of hardness (Figure 3b). The results showed that the CH-PE coating reduced the acting substrate of PG by inhibiting PME activity during storage, thereby reducing the hydrolysis of pectin and maintaining the hardness of the mangoes. Ma et al. [28] reported that the composite coating based on shellac and an active agent, tannic acid, slowed down the hydrolysis of pectin and retained the mangoes’ hardness by inhibiting the PG activity of the mangoes. Another study suggested that NO + wax treatment delayed the peak of PG activity in mangoes for 4 d at 13 °C, which was positively correlated with hardness [20].

## 4. Conclusions

The chitosan (CH)-cinnamon essential oil (CEO) Pickering emulsion (CH-PE) coating was prepared, and had a lower water solubility and water vapor permeability than the pure CH coating and CH-CEO emulsion (CH-E) coatings that were prepared with Tween 80, mainly due to the electrostatic interactions. Moreover, the CH-PE coating can delay the release of CEO more effectively in different food simulants than the CH-E coating. Furthermore, the CH-PE coating was applied to mangoes, which were stored at 25 °C for 12 d. For uncoated mangoes, the peels turned yellow rapidly and large areas of dark spots appeared during storage. After 12 d of storage, the hardness, titratable acidity and ascorbic acid content decreased to 70.792 g, 0.074% and 14.42 mg/100 g, respectively, and the water loss increased to 24.437%. The activities of PPO, PME and PG, as well as content of MDA, were relatively high, and the activity of POD was relatively low. However, the CH-PE coating treatment reduced the enzymatic browning of mangoes by inhibiting the activity of PPO, decreased water loss, and delayed the decrease of mango hardness by inhibiting the activities of PME and PG. Moreover, the CH-PE coating improved nutrient quality by increasing the TAA, titratable acid, and ascorbic acid content, as well as reduced the accumulation of MDA content by promoting the increase of POD activity. The fresh-keeping effect of the CH-PE coating on mangoes was obviously better than those of the CH and CH-E coatings. Especially, the activity of disease-resistant enzyme PAL in mangoes was not significantly affected and activated by CH-PE coating due to less disease incidence. The research results showed that the CH-PE coating is an ideal choice for prolonging the shelf life of harvested mangoes, and the coating has a wide range of applications in the food industry.

## Figures and Tables

**Table 1:** Zeta-potential of cinnamon essential oil (CEO) emulsion (E), CEO Pickering emulsion (PE), chitosan (CH) solution, CH-CEO emulsion (CH-E) and CH-CEO Pickering emulsion (CH-PE), and water solubility (WS) and water vapor permeability (WVP) of CH, CH-E and CH-PE coatings.

	E	PE	CH	CH-E	CH-PE
Zeta-potential (mV)	19.167 ± 0.551 ^c^	−55.133 ± 3.844 ^d^	34.033 ± 4.255 ^b^	67.367 ± 6.882 ^a^	56.000 ± 7.672 ^a^
WVP (×10^−11^ g/m·s·Pa)	-	-	11.869 ± 0.379 ^a^	7.518 ± 0.724 ^b^	5.222 ± 0.100 ^c^
WS (%)	-	-	31.829 ± 0.438 ^a^	17.463 ± 0.797 ^b^	13.911 ± 0.985 ^c^

^a–d^ Different superscript lowercase letters indicate statistical significance (*p* < 0.05).

## Data Availability

The datasets generated for this study are available on request to the corresponding author.
